# *Socs3* expression in myeloid cells modulates the pathogenesis of dextran sulfate sodium (DSS)-induced colitis

**DOI:** 10.3389/fimmu.2023.1163987

**Published:** 2023-05-22

**Authors:** Lianna Zhou, Zhaoqi Yan, Wei Yang, Jessica A. Buckley, Sameer Al Diffalha, Etty N. Benveniste, Hongwei Qin

**Affiliations:** ^1^ Department of Cell, Developmental and Integrative Biology, University of Alabama at Birmingham, Birmingham, AL, United States; ^2^ Gladstone Institute of Neurological Disease, San Francisco, CA, United States; ^3^ Division of Gastroenterology and Hepatology, Weill Cornell College of Medicine, New York, NY, United States; ^4^ Department of Pathology, University of Alabama at Birmingham, Birmingham, AL, United States

**Keywords:** inflammatory bowel diseases (IBDs), ulcerative colitis (uc), suppressors of cytokine signaling (SOCS), myeloid cells, neutrophils

## Abstract

**Introduction:**

Myeloid cells play a critical role in the pathogenesis of Inflammatory Bowel Diseases (IBDs), including Ulcerative Colitis (UC) and Crohn’s Disease (CD). Dysregulation of the JAK/STAT pathway is associated with many pathological conditions, including IBD. Suppressors Of Cytokine Signaling (SOCS) are a family of proteins that negatively regulate the JAK/STAT pathway. Our previous studies identified that mice lacking *Socs3* in myeloid cells developed a hyper-activated phenotype of macrophages and neutrophils in a pre-clinical model of Multiple Sclerosis.

**Methods:**

To better understand the function of myeloid cell *Socs3* in the pathogenesis of colitis, mice with *Socs3* deletion in myeloid cells (*Socs3* ^ΔLysM^) were utilized in a DSS-induced colitis model.

**Results:**

Our results indicate that *Socs3* deficiency in myeloid cells leads to more severe colitis induced by DSS, which correlates with increased infiltration of monocytes and neutrophils in the colon and increased numbers of monocytes and neutrophils in the spleen. Furthermore, our results demonstrate that the expression of genes related to the pathogenesis and diagnosis of colitis such as *Il1β*, *Lcn2*, *S100a8* and *S100a9* were specifically enhanced in *Socs3*-deficient neutrophils localized to the colon and spleen. Conversely, there were no observable differences in gene expression in Ly6C^+^ monocytes. Depletion of neutrophils using a neutralizing antibody to Ly6G significantly improved the disease severity of DSS-induced colitis in *Socs3*-deficient mice.

**Discussion:**

Thus, our results suggest that deficiency of *Socs3* in myeloid cells exacerbates DSS-induced colitis and that *Socs3* prevents overt activation of the immune system in IBD. This study may provide novel therapeutic strategies to IBD patients with hyperactivated neutrophils.

## Introduction

Inflammatory Bowel Diseases (IBDs) are chronic relapsing inflammatory disorders of the gastrointestinal tract, including two major subtypes: Ulcerative Colitis (UC) and Crohn’s Disease (CD) (1, 2). IBD has drawn wide attention with a high rate of prevalence in the Western world and East Asia with connections to the Westernized lifestyle and environmental factors such as smoking, microorganisms, medication, nutrition, and stress (3, 4). Both UC and CD share common symptoms and structural damages, which are associated with multiple pathogenic factors including environmental changes, susceptibility gene variants, and a broadly dysregulated immune response (5, 6). A comprehensive understanding of IBD pathogenesis is still unclear and, consequently, treatment is far from optimal. One of the animal models used to investigate the molecular and cellular mechanisms of IBD is the acute dextran sodium sulfate (DSS) colitis model, which is frequently used to develop and evaluate the efficacy of novel anti-inflammatory drugs (7). The acute inflammatory responses in the DSS colitis model are particularly useful to study the contribution of innate immune mechanisms in intestinal inflammation (8, 9).

The JAK/STAT signaling pathway plays a critical role in activation and regulation of immune responses (10, 11). Dysregulation of the JAK/STAT pathway is associated with many pathological conditions, including Multiple Sclerosis (MS), Rheumatoid Arthritis and IBDs (12, 13). We previously demonstrated that inhibition of the JAK/STAT pathway ameliorates disease severity in a number of pre-clinical models of MS and Parkinson’s disease (14, 15). There is emerging evidence that the JAK/STAT pathway has pathogenic roles in the development of colitis; JAK inhibitors, such as tofacitinib, have been approved for treatment of UC with demonstrated efficacy in early phases of clinical trials (16, 17). As a family of proteins that negatively regulate the JAK/STAT pathway, Suppressors Of Cytokine Signaling 3 (SOCS3) mainly inhibits activation of STAT1 and STAT3 (18, 19). Studies from animal models, including the DSS-induced colitis model, have established that STAT3 activation promotes pathogenesis of UC and carcinogenesis (20–25). However, other results indicate STAT3 deletion in myeloid cells also leads to exacerbation of colitis (26, 27). As such, further study is required to elucidate the role of the STAT3/SOCS3 axis in myeloid cells to understand their role in the pathogenesis of colitis.

The role of myeloid cells in IBD is also inconclusive. Monocytes and macrophages play pivotal roles in the innate immune response to pathogens primarily through phagocytosis and the release of inflammatory mediators such as cytokines and chemokines (28). Previous studies have shown that under inflammatory conditions, monocytes migrate out of the blood utilizing a CCR2-related mechanism and are recruited to the lamina propria, thereby favoring a pro-inflammatory response (29–32). Macrophage- and monocyte-related pro-inflammatory cytokines such as TNF, IL-1β, IL-6 and IL-1 are detected in colonic biopsies from patients with CD and UC and are implicated in the development of IBD in humans (33). Moreover, inhibition of monocyte/macrophage recruitment led to ameliorated murine colitis (34). Neutrophils are the most abundant cell type in human blood and are the first responders of the immune system when the body encounters foreign antigens (35, 36). Neutrophil infiltration into the lamina propria is an important histological activity index in UC (37) and promotes disease progression in animal models of colitis (38). However, other studies demonstrated that bone marrow-derived suppressor cells, which consist of macrophages and neutrophils, suppress overt inflammation, accelerate recovery, and protect the epithelial barrier in the intestine (39–41).

We generated a mouse model, crossing *Socs3* ^fl/fl^ mice with LysMCre mice, to create a conditional knockout mouse possessing *Socs3*-deficient myeloid cells (*Socs3* ^ΔLysM^) (42, 43). Previous studies in our lab using *Socs3* ^ΔLysM^ mice demonstrated enhanced basal and stimulus-induced STAT activation, in addition to upregulation of various pro-inflammatory mediators in *Socs3*-deficient myeloid cells (42, 43). Furthermore, both *Socs3*-deficient macrophages and neutrophils displayed gene expression patterns reflective of hyper-activated cells with pro-inflammatory properties (43, 44). These pro-inflammatory responses were observed in animal models of MS and LPS-induced sepsis (42, 43). Overall, these studies demonstrate that deletion of *Socs3* in myeloid cells contributes to hyper-activation and enhanced inflammation, leading us to postulate that *Socs3-*deficient myeloid cells may play a potential role in the pathogenesis of DSS-induced colitis.

In this study, we investigated the pathogenic function of myeloid cells in a colitis model induced by DSS in *Socs3* ^ΔLysM^ mice. Our results demonstrate that neutrophils from *Socs3* ^ΔLysM^ mice are critical for disease severity and inflammatory responses in DSS colitis. Furthermore, neutrophil depletion using neutralizing antibody 1A8 significantly reduces inflammation in DSS colitis in *Socs3* ^ΔLysM^ mice. Overall, our findings demonstrate the importance of *Socs3* in repressing the hyper-activation of myeloid cells, specifically neutrophils, which contribute to our understanding of the role of neutrophils in IBD/colitis.

## Materials and methods

### Mice

All animal experiments were reviewed and approved by the Institutional Animal Care and Use Committee of UAB. C57BL/6 mice were bred in an animal facility at the University of Alabama at Birmingham (UAB). Transgenic mice with the *Socs3* locus flanked with flox sequences [*Socs3* ^fl/fl^; (45)] were the generous gift of Dr. Warren Alexander (Walter and Eliza Hall Institute of Medical Research; Victoria, Australia) and were bred at UAB. *Socs3* conditional knockout (*Socs3* ^ΔLysM^) mice were generated by serial breeding of *Socs3* ^fl/fl^ mice with mice expressing Cre-recombinase under the control of the *LysM* promoter (42, 44). All mice were used at 8-12 weeks of age.

### DSS-induced colitis model and disease severity scores

Colitis was induced in female *Socs3* ^fl/fl^ and *Socs3* ^ΔLysM^ mice with 3% DSS (molecular weight=40 kDa, MP Biomedical. Solon, OH), which was supplied in drinking water as described (8). DSS-induced colitis in male mice shows higher severity and faster progression compared to female mice. Combining both sexes significantly increased variability, therefore only female mice were used in this study. Weight loss of mice was monitored daily, and mice were sacrificed at day 5 for immune cell analysis and at day 7 for histology analysis (8, 46, 47). A scoring system was applied to assess diarrhea and the presence of occult or overt bleeding in the stool. Disease severity scores were calculated at day 7 by weight loss, stool bleeding, and stool consistency, with combined scores ranging from 0 to 12 (**Table 1**). Disease severity scores were determined by two investigators blinded to the treatment groups.

**Table 1:** DSS disease severity scoring criteria.

Scores	Weight Loss	Stool Blood	Stool Consistency
0	0% - 1%	Negative	Normal
1	1%-5%		
2	6% - 10%	Positive	Loose Stools
3	11% - 15%		
4	>15%	Gross Bleeding	Diarrhea

Disease severity was scored daily, 0 - 4, using severities of weight loss, stool blood and stool consistency.

### Preparation of lamina propria immune cells

Colon lamina propria cells were prepared using the Lamina Propria Dissociation Kit (Miltenyi Biotec, Auburn, CA) according to the manufacturer’s instructions (48). Lamina propria immune cells were from the intestines as previously described (49). Tissues were rinsed, sliced into small pieces, and incubated at 37°C for 40 min. The tissues were then digested for 50 min at 37°C by continuous stirring with magnetic micro stir bars. The liberated cells were collected by passage through a 100 μm cell strainer (BD Falcon). Mononuclear cells were isolated from the lamina propria using a 40%/75% Percoll (GE Healthcare, 17089101) gradient. The isolated cells were resuspended in R10 medium (RPMI 1640 with 10% FBS, 2 mM L-glutamine, 100 U/ml penicillin, 100 μg/ml streptomycin, 10 mM HEPES, 1 mM sodium pyruvate, and 50 μM β-mercaptoethanol). The cell yield was typically 2-3 x 10^6^ immune cells per mouse with 90% cell viability.

### Splenocyte preparation

Single-cell suspensions of spleen were prepared as previously described (50, 51) and resuspended in R10 medium.

### Histological analysis

Swiss rolls of colons were prepared as previously described (52). Tissues were fixed in 10% formalin in water overnight prior to paraffin-embedding. Paraffin-embedded samples were cut into 5 μm sections followed by hematoxylin and eosin (H&E) staining, and histopathology was evaluated in a double-blinded manner by a GI pathologist. Epithelium damage and inflammation severity were scored separately, and a total score was given accordingly. These scores were comprised of the following factors: severity of lesion, degree of hyperplasia, degree of ulceration, and percent of area involved. Severity of lesion referred to the overall size, quantity, and spatial dispersion of lesions. Degree of hyperplasia referred to crypt and goblet cell morphology, hyperchromasia, and mitotic index of goblet cells. Degree of ulceration referred to number of ulcers and the number of crypts involved. Epithelial damage was evaluated in all sections by hyperplasia, goblet cell loss, degeneration and necrosis, ulceration, and dysplasia. Inflammation severity was evaluated by crypt exudate, lamina propria and submucosal inflammatory cell accumulation intensity, submucosal edema distribution and transmural inflammation. Severity of lesion was graded as follows: 0 = normal, 1 = mild, 2 = moderate, 3 = severe (7, 48, 53).

### Antibodies and flow cytometry

For flow cytometry experiments, antibodies (Abs) directed against murine CD11b (M1/70), CD45 (30-F11), Ly6C (HK1.4), Ly6G (1A8), CD3 (17A2), CD4 (GK1.5), and CD19 (6D5) were from BioLegend. Cell phenotypes were determined based on surface staining patterns analyzed by flow cytometry, as previously described (14, 42, 44, 48). All flow cytometry data were analyzed using FlowJo software (BD), as previously described (44). For neutrophil depletion experiments, Ly6G was detected after by intracellular staining of Ly6G after fixation and permeablization with eBioscience™ Foxp3/Transcription Factor Staining Buffer Set (Invitrogen, 00-5523-00) (44).

### RNA isolation and quantitative RT-PCR

Total RNA was purified from FACS-sorted neutrophils and Ly6C^+^ monocytes isolated by flow cytometry using TRIzol reagent extraction. For qRT-PCR analysis, 500-1000 ng of RNA was used as a template for cDNA synthesis (Promega). qRT-PCR was performed using TaqMan primers purchased from Thermo Fisher Scientific. The resulting data were analyzed using the comparative cycle threshold method to calculate relative RNA quantities (44, 50).

ELISA Analysis. Fecal samples were collected from day 4 post-DSS administration. LCN2 levels were measured by ELISA using the Lipocalin-2 (LCN2) Mouse ELISA Kit (ThermoFisher) (8, 54).

### Statistics

Significant differences between two groups were analyzed by the nonparametric Mann-Whitney test. One-way ANOVA was used to compare differences between more than two variables. *P*-values less than 0.05 were considered statistically significant. All error bars represent standard error of the mean (SEM).

## Results

### *Socs3* deficiency in myeloid cells promotes colitis disease development

**Figure 1:**
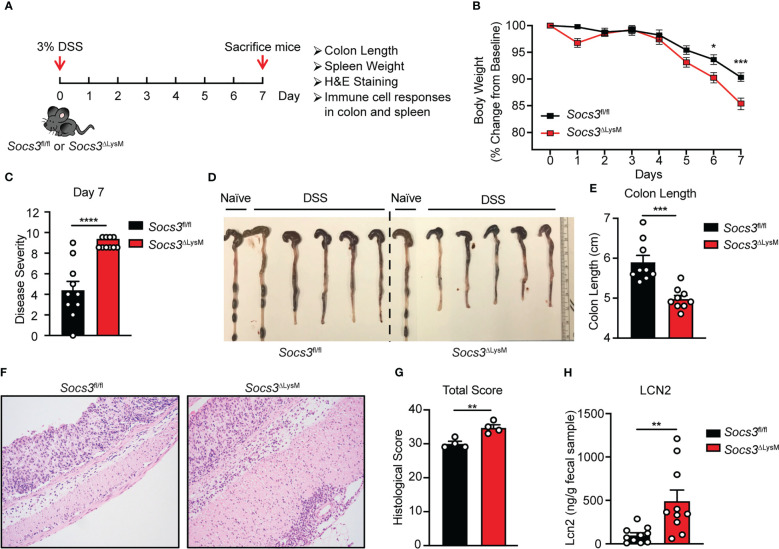
*Socs3* deficiency in myeloid cells leads to more severe colitis induced by DSS. **(A)** Female *Socs3* ^fl/fl^ (n = 10) or *Socs3* ^ΔLysM^ (n = 10) mice were fed with 3% DSS for 7 days. Mice were weighed daily, and weight loss was determined by comparing to weight on Day 0. Mice were euthanized on day 7. Colon and spleen were collected for colon length, spleen weight, colon histology and immune cell responses. **(B)** Mice were weighed daily, and weight loss was determined by comparing to weight on Day 0. **(C)** Disease severity was calculated on day 7. **(D)** Representative pictures of the colon. **(E)** Statistics of colon length (n = 9). **(F)** Haemotoxylin and Eosin **(H&E)** staining of the distal colon. Pictures are representation of 4 mice. **(G)** Total pathological scores of colons are shown using severity of lesion, degree of hyperplasia, degree of ulceration and percent of area involved (n = 4) in *Socs3* ^fl/fl^ or *Socs3* ^ΔLysM^ mice. **(H)** Fecal samples were collected on day 4 post-DSS administration. LCN2 levels were determined by ELISA (n = 10). *p < 0.05, **p<0.01, ***p < 0.001, ****p < 0.0001.

To assess the function of *Socs3* in myeloid cells in the pathogenesis of colitis, *Socs3* ^fl/fl^ mice crossed with LysM-Cre mice (*Socs3* ^ΔLysM^), which leads to deletion of *Socs3* in myeloid cells, including macrophages, neutrophils and partially in dendritic cells, were used (43, 44). Acute colitis was induced in mice by administering water containing 3% DSS polymers for 7 days (**Figure 1A**), which induces a colitis characterized by diarrhea, bloody feces, and weight loss as seen in human IBD. Results demonstrate that *Socs3* ^ΔLysM^ mice have more severe colitis compared to *Socs3* ^fl/fl^ mice at days 6 and 7 after DSS feeding. *Socs3* ^ΔLysM^ mice exhibited reduced body weight, increased disease severity scores, and reduced colon lengths compared to *Socs3* ^fl/fl^ mice (**Figures 1B–E**). Colon length was recorded as a proxy for degree of longitudinal contraction as a result of inflammation, diarrhea, and hypertrophy of the muscularis mucosae (55). Consistent with these findings, histopathological analysis showed more severe inflammation in the colon of *Socs3* ^ΔLysM^ mice compared to *Socs3* ^fl/fl^ mice (**Figures 1F, G**). Fecal lipocalin 2 (LCN2), measured by ELISA, is a non-invasive and well-characterized biomarker of intestinal inflammation in the DSS colitis model (8, 54). ELISA was performed on fecal samples collected from day 4 post-DSS administration due to difficulties in sample collection on later days. LCN2 levels were significantly higher in the fecal samples from *Socs3* ^ΔLysM^ mice compared to *Socs3* ^fl/fl^ mice at day 4 (**Figure 1H**). Colon length, colon histology, spleen weight, and distribution of immune cell populations in the colon and spleen from naïve *Socs3* ^fl/fl^ and *Socs3* ^ΔLysM^ mice were comparable (**Supplementary Figure 1**). These results indicate that *Socs3* expression in myeloid cells is required for suppressing the development of DSS-induced colitis.

### *Socs3* deficiency increases infiltration of innate immune cells in the colon of DSS colitis mice

**Figure 2:**
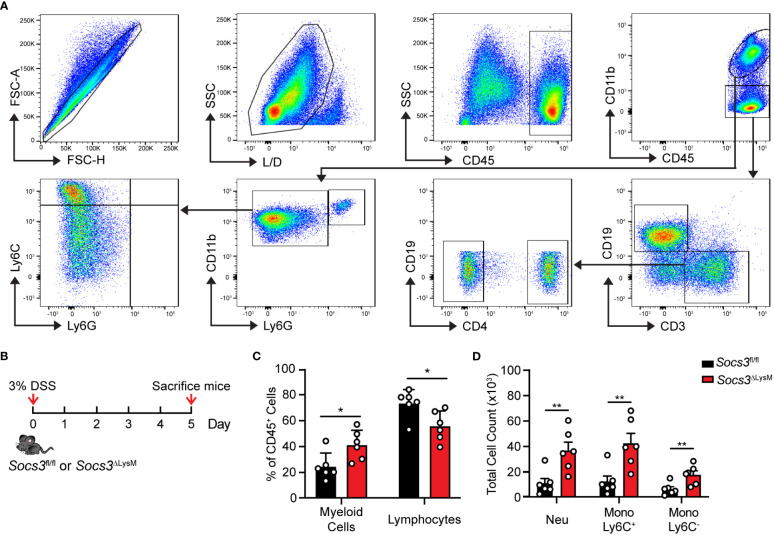
*Socs3* deficiency increases colonic myeloid cell infiltration induced by DSS. **(A)** Singlets were gated for live CD45^+^ immune cells, CD45^+^CD11b^+^ myeloid cells and CD45^+^CD11b^-^ lymphocytes. Ly6G^+^ neutrophils and Ly6G^-^ monocytes were gated from the myeloid population; CD19^-^CD4^+^ T-cells, CD19^-^CD4^-^ T-cells and CD3^-^CD19^+^ B-cells were gated from lymphocytes. Ly6C^+^ monocytes and Ly6C^-^ monocytes were gated from the Ly6G^-^ population. **(B)** *Socs3* ^fl/fl^ (n = 6) or *Socs3* ^ΔLysM^ (n = 6) mice were fed with 3% DSS. Mice were euthanized on day 5 and colonic infiltrating cells were isolated and stained for multicolor flow cytometry analysis. Total number of infiltrating myeloid cells and lymphocytes **(C)** and neutrophils and different monocyte subsets **(D)** in the colon from *Socs3* ^fl/fl^ or *Socs3* ^ΔLysM^ mice were calculated and compared. not significant (ns) p ≥ 0.05, *p < 0.05, **p < 0.01 and ***p<0.001.

To elucidate the primary cell type(s) responsible for the inflammation observed in this model, we used multicolor flow cytometry analysis to observe changes in cell populations. We characterized immune cell infiltration when weight loss was apparent and signs of inflammation were macroscopically visible, specifically day 5 post-DSS administration (**Figure 2B**). Immune cell populations were determined and assessed using the gating strategy represented in **Figure 2A**. As a percentage of total CD45^+^ cells, *Socs3* ^ΔLysM^ mice displayed a significant increase in CD45^+^CD11b^+^ myeloid cells and a significant decrease in the CD45^+^CD11b^-^ lymphocyte population (**Figure 2C**). As a measure of total cell counts, *Socs3* ^ΔLysM^ mice displayed significantly increased CD45^+^CD11b^+^Ly6G^+^ neutrophil (Neu), Ly6G^-^Ly6C^+^ monocyte (Mono Ly6C^+^) and Ly6G^-^Ly6C^-^ monocyte (Mono Ly6C^-^) populations (**Figure 2D**). Lymphocytes were also further characterized into CD4^+^ and CD4^-^ T-cell subsets and B-cells, with no differences observed in any population in the colon (**Supplementary Figure 2A**). These results suggest that myeloid cells, specifically neutrophils, Ly6C^+^ monocytes and Ly6C^-^ monocytes, may have a potential role in the hyper-inflammation observed in DSS-induced colitis in *Socs3* ^ΔLysM^ mice.

### *Socs3* deficiency in myeloid cells increases infiltration of innate immune cells in the spleen of DSS colitis mice

**Figure 3:**
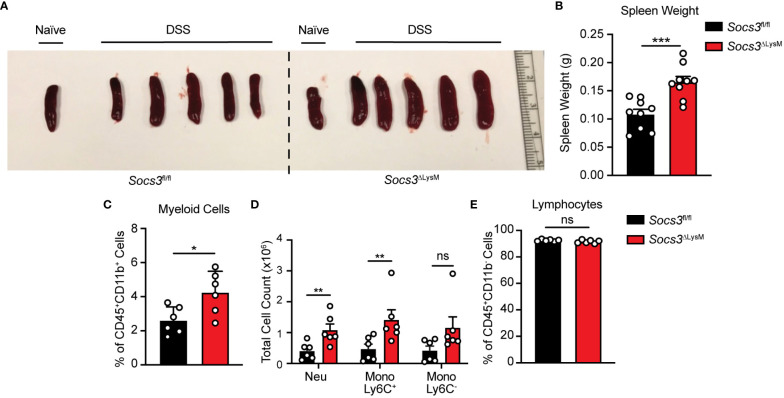
*Socs3* deficiency increases splenic myeloid cell numbers in DSS-induced colitis. Representative spleen picture **(A)** and statistics of spleen weight **(B)** at day 7 (n = 9). Splenocytes were isolated and stained at day 5 as described in **Figures 2A, B**. Percentage of CD45^+^CD11b^+^ myeloid cells **(C)** and cell numbers of neutrophils and different monocyte subsets **(D)** from *Socs3* ^fl/fl^ (n = 6) or *Socs3* ^ΔLysM^ (n = 6) mice fed with 3% DSS on day 5. Percentage of CD45^+^CD11b^-^ lymphocytes (n = 6) **(E)** in the spleen from *Socs3* ^fl/fl^ or *Socs3* ^ΔLysM^ mice. *p < 0.05, ***p < 0.001.

In accordance with our findings of immune cells derived from the colon, we wanted to confirm whether these were mirrored in systemic inflammation as represented by splenic size and weight. In correlation with colon lengths depicted in **Figures 1D, E**, we first report splenic weight after 7 days of DSS administration and found that regardless of mouse phenotype, both groups of mice developed splenomegaly as compared to spleens taken from healthy controls (**Figure 3A**). However, mice lacking *Socs3* displayed an exacerbated effect, having significantly larger spleens than those with intact *Socs3* (**Figure 3B**). Because the overall increase in size and weight suggested an increase in immune cell infiltration, we further characterized isolated splenocytes at our designated early disease time point, day 5, using multicolor flow cytometry. Using the same gating strategy from **Figure 2**, we found results comparable to those from isolated colonic cells. *Socs3* ^ΔLysM^ mice not only displayed a significant increase in overall myeloid cells, but this increase was observed in the Neu and Mono Ly6C^+^ populations but not the Mono Ly6C^-^ population (**Figures 3C, D**). While there was no difference between the overall lymphocyte population (**Figure 3E**), a significant increase in the CD4^+^ T-cell subpopulation in the spleen was observed, but not in the CD4^-^ T-cell subpopulation nor the B-cell population (**Supplementary Figure 2B**). Overall, these data indicate that *Socs3* deficiency leads to increased inflammatory responses in the colon and spleen of these mice.

### Proinflammatory mediators are enhanced in *Socs3*-deficient neutrophils from the colon of DSS colitis mice

**Figure 4:**
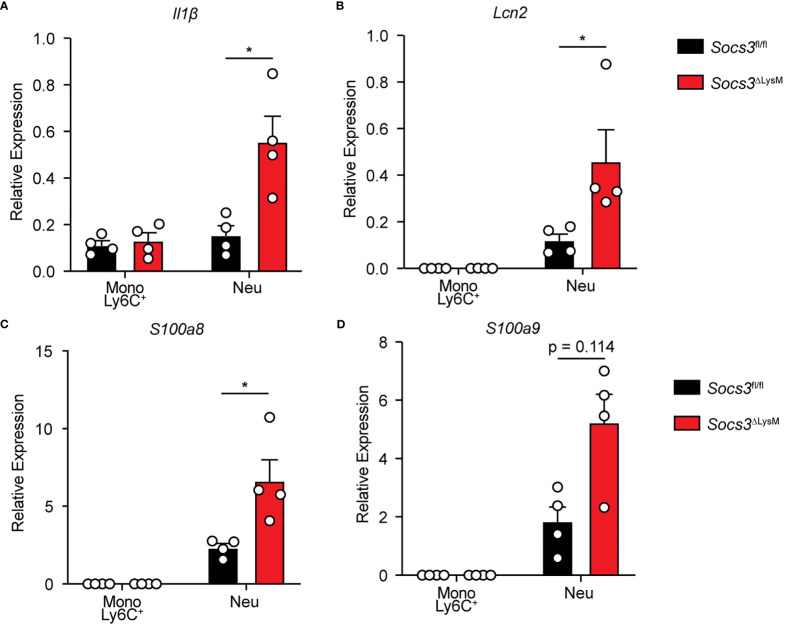
*Socs3*-deficient neutrophils produce increased proinflammatory mediators in DSS-induced colitis. *Socs3* ^fl/fl^ (n = 8) or *Socs3* ^ΔLysM^ (n = 8) mice were fed with 3% DSS. Mice were euthanized on day 5, and immune cells were sorted from colon tissue by flow cytometry. Gene expression in Ly6C^+^ monocytes and neutrophils from *Socs3* ^fl/fl^ or *Socs3* ^ΔLysM^ mice was examined. Gene expression of *Il1β* **(A)**, *Lcn2* **(B)**, *S100a8* **(C)**, and *S100a9* **(D)** were determined by qRT-PCR, using 18s rRNA as an internal control. Two mice were combined for each experiment with 4 experiments in each group. *p < 0.05.

Considering that *Socs3* ^ΔLysM^ mice displayed an increase in both monocyte and neutrophil populations, we compared the expression of proinflammatory mediators at the RNA level through qRT-PCR using sorted Ly6C^+^ monocytes and neutrophils from colonic tissue. Previous studies have suggested that Ly6C^hi^ monocytes are precursors to macrophages during inflammatory conditions while Ly6C^lo^ monocytes are precursors to steady-state tissue macrophages (56–58). Therefore, these experiments were performed using Ly6C^+^ monocytes instead of Ly6C^-^ monocytes. Our results indicated that colonic neutrophils from *Socs3* ^ΔLysM^ mice exhibited higher expression levels of *Il1β* compared to those of *Socs3* ^fl/fl^ mice (**Figure 4A**). Additionally, we examined changes in expression of genes pertinent to the pathogenesis of colitis. We found that neutrophils from *Socs3* ^ΔLysM^ mice displayed higher levels of expression of *Lcn2, S100a8*, and *S100a9* (**Figures 4B–D**), with the latter two having emerging roles as pre-clinical biomarkers for neutrophilic inflammation such as that observed in IBDs (39, 53, 54). Interestingly, there were no observable differences between *Socs3* ^fl/fl^ and *Socs3* ^ΔLysM^ Ly6C^+^ monocyte populations for any of these genes (**Figures 4A–D**). Additional genes reportedly expressed by monocytes/macrophages, including *Cd74, Tnfα, Il6, Il12α, Il12β, Ccl2, Cxcl1, Cxcl10 and iNOS*, were determined by qRT-PCR (**Supplementary Figure 3**) (42, 43, 59). Interestingly, the only significant difference we found was that *Socs3* ^ΔLysM^ neutrophils displayed significantly higher levels of *Il12β* expression compared to *Socs3* ^fl/fl^ neutrophils. These findings suggest that monocytes/macrophages may not be the culprit of the heightened colonic inflammation in *Socs3* ^ΔLysM^ mice. Rather, *Socs3*-deficient neutrophils are critical for the exacerbation of DSS-induced colitis in *Socs3* ^ΔLysM^ mice.

### Depletion of neutrophils significantly improves the development of DSS colitis in *Socs3* ^ΔLysM^ mice

**Figure 5:**
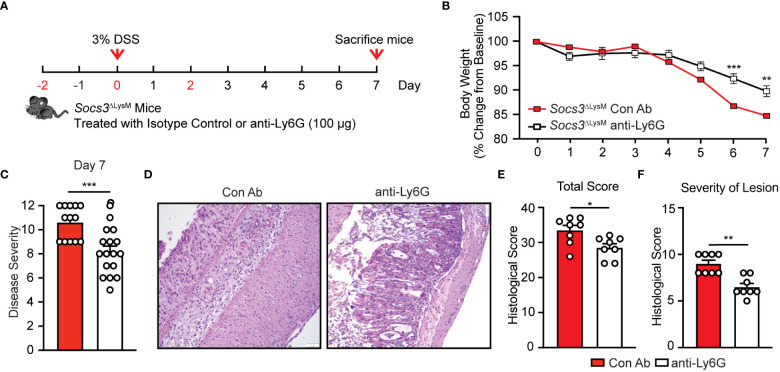
Neutrophil depletion significantly improves DSS-induced colitis in *Socs3* ^ΔLysM^ mice. **(A)** Female *Socs3* ^ΔLysM^ mice were injected with either anti-Ly6G (anti-Ly6G; n = 17) or Isotype Control (Con Ab; n = 13) Abs (100 µg/dose) intraperitoneally at days -2, 0 and 2 (shown in red). Mice were fed with 3% DSS for 7 days. **(B)** Anti-Ly6G (n = 17) and Con Ab (n = 13) mice were weighed daily, and weight loss was determined by comparing to weight daily from day 0 to day 7. **(C)** Disease severity on day 7. **(D)** H&E staining of the distal colon. Pictures are representation of 8 mice. **(E)** Total pathological scores of colons are shown using severity of lesion, degree of hyperplasia, degree of ulceration and percent of area involved (n = 8). **(F)** Severity of lesion was calculated in *Socs3* ^ΔLysM^ mice administered anti-Ly6G or isotype control abs. *p < 0.05, **p < 0.01 and ***p<0.001.

Thus far, our results have suggested a strong correlation between *Socs3* deletion, augmented neutrophil infiltration, increased expression of pro-inflammatory mediators by neutrophils, and exacerbated colitis. We, therefore, hypothesized that depletion of neutrophils would ameliorate intestinal inflammation and mitigate the effects of DSS colitis in *Socs3* ^ΔLysM^ mice. To determine the role of neutrophils in the exacerbation of DSS-induced colitis in *Socs3* ^ΔLysM^ mice, we developed an acute neutrophil depletion regimen by injection of anti-Ly6G antibody. Our method results in a significantly lower frequency of circulating neutrophils for seven days in the anti-Ly6G group compared to the isotype control Ab group (**Supplementary Figure 4**), confirming successful and conserved neutrophil depletion. We depleted neutrophils two days prior to DSS administration, followed by one dose on the initial day of DSS administration, and a final dose two days thereafter (**Figure 5A**). After seven days of DSS administration, mice were euthanized for spleen and colon tissue collection. Importantly, *Socs3* ^ΔLysM^ mice that received the neutrophil depletion antibody exhibited less severe forms of DSS-induced colitis as indicated by reduced body weight loss and lower disease severity scores (**Figures 5B, C**). To confirm our findings at the histopathological level, we stained the colonic tissue using H&E staining for imaging to assess and quantify the degree of intestinal damage. As shown in the representative images, we found that neutrophil depletion led to partial conservation of tissue structure, which was supported by significantly lower total histological scores (**Figures 5D, E**). Mice treated with the anti-Ly6G antibody displayed significantly lower severity of lesion scores compared to those that received the isotype control (**Figure 5F**).

To determine whether these therapeutic effects could be recapitulated in *Socs3* ^fl/fl^ mice, these mice were subjected to the same neutrophil-depleting treatment as the *Socs3* ^ΔLysM^ mice. Neutrophil depletion had no effect on DSS-induced colitis in *Socs3* ^fl/fl^ mice. As shown in **Supplementary Figure 5**, weight loss, disease severity scores, and histological analysis at day 7 were comparable between the anti-Ly6G treated mice and the isotype control Ab treated mice. Taken together, these data suggest the detrimental role of hyperactivated neutrophils in the pathogenesis of DSS-induced colitis in *Socs3* ^ΔLysM^ mice.

## Discussion

In the present study, we report the detrimental role of myeloid cell *Socs3* deletion in DSS-induced colitis. We performed unbiased FACS analysis and discovered that *Socs3* deficiency in myeloid cells leads to profound infiltration of macrophages and neutrophils in the lamina propria. Interestingly, only neutrophils from *Socs3* ^ΔLysM^ mice show increased pro-inflammatory properties, compared to *Socs3* ^fl/fl^ controls. Furthermore, depleting *Socs3*-deficient neutrophils using a monoclonal neutralizing antibody significantly improved the pathology of colitis in mice. Our findings support the critical role of SOCS3, a negative regulator of the JAK/STAT pathway, in neutrophils for mitigating intestinal inflammation in a DSS-induced model of IBD.

To date, there is no evidence indicating that *Socs3* is a risk factor for IBD. However, recent studies have shown that *Socs3* mRNA expression is increased in the mucosa tissue of UC patients, compared to inactive control tissues (60). The same study also reported reduced *Socs3* in IBD patients to be correlated with more frequent remission (60). Another study supported these findings by identifying high levels of SOCS3 protein in UC patients (61). Conversely, other groups found lower *Socs3* expression to be associated with IBD-associated dysplasia (62) as well as an increased susceptibility to IBD (63). These findings demonstrate the complex role of *Socs3* in IBD, suggesting context-dependent functionality, either beneficial or detrimental.

While the original objective of this study was to focus on myeloid cells, our data unveiled a detrimental role of neutrophils in an animal model of IBD. Although this is in line with some reports (38), other studies suggest that neutrophil depletion exacerbates intestinal inflammation (39, 64, 65). This discrepancy in findings can be explained by the ever-growing importance of neutrophil heterogeneity as well as neutrophil subsets. Historically, neutrophils were thought to be a homogeneous population of terminally differentiated cells. However, recent studies have shown that neutrophils display phenotypic and transcriptional plasticity and diversity in terms of functionality (66, 67). The occurrence of neutrophil heterogeneity has recently been confirmed, and phenotypic clusters have been characterized by scRNA-Seq (68–70). More importantly, these neutrophil subsets play opposite roles during intestinal inflammation. Recent studies using scRNA-Seq revealed complex heterogeneity of neutrophils in homeostasis and inflammation (71, 72) and demonstrated dual roles of neutrophils during inflammation. Future studies will use scRNA-Seq to examine the composition of neutrophils in animal models of IBD and determine whether hyperactive JAK/STAT signaling led by *Socs3* deficiency skews neutrophil subsets towards a pro-inflammatory status.

Our current study revealed significant up-regulation of antimicrobial peptides in *Socs3*-deficient neutrophils during inflammation, namely LCN2, S100A8, and S100A9. The role of antimicrobial peptides is also complex. LCN2 promotes neutrophil-mediated anti-bacterial activity (73), and the serum level of LCN2 is an ideal biomarker of active UC (74). However, *Lcn2* deficiency leads to severe intestinal inflammation in *Il10* ^-/-^ mice (75), indicating a complex role for *Lcn2* in IBDs. S100A8/S100A9 are also sensitive biomarkers for IBD (76) and work as endogenous activators of Toll-like receptor 4 (TLR4) (77). On the other hand, S100A8/S100A9 are also critical to contain inflammation at the local level, as S100A9^-/-^ mice result in TNF-α-driven fatal inflammation (78). Future studies will determine the role of increased anti-microbial peptides in *Socs3*-deficient neutrophils in colitis.

In addition to antimicrobial peptides, neutrophils have been shown to increase production of pro-inflammatory cytokines and chemokines during intestinal inflammation (79). Cytokines such as IL-1β, IL-6, IL-8, TNF-α, GM-CSF and G-CSF are critical for secondary neutrophil recruitment and infiltration into the intestinal mucosa, and chemokines such as CXCL-1, -8, and -10 are necessary for recruitment of other immune cells (80). Our study revealed an increase in *Il1β* expression by *Socs3*-deficient neutrophils at the RNA level during DSS-induced colitis. This result is congruent with clinical findings of higher IL-1β concentrations present in the intestines of IBD patients (81). The same study described crosstalk between IL-1β and IL-23 to sustain both innate and adaptive inflammatory responses during intestinal inflammation (81). A recent study also demonstrated that a subset of IBD patients, non-responsive to anti-TNF, corticosteroid, and anti-integrin therapy, showed significant IL-1β*-*driven neutrophil interaction with intestinal stromal cells (82). Future studies will perform comprehensive profiling to observe changes in a broader selection of pro-inflammatory mediators, including cytokines (e.g., IL-1β, G-CSF), chemokines (e.g., CXCL1, CXCL10) and oxidative stress-related free radicals, as a result of *Socs3* deletion.

Our data also suggest that inhibiting neutrophil activity attenuates experimental colitis as neutrophil depletion significantly improved disease in the DSS colitis model in *Socs3* ^ΔLysM^ mice. However, because these findings were not observed in *Socs3* ^fl/fl^ mice, further studies on the potential protective mechanisms of *Socs3* in human IBD development are needed. Additionally, the exact source of neutrophils’ deleterious effects is unclear. Some studies report impairment of neutrophil recruitment and trafficking significantly improving the pathology of colitis (83, 84), while others have demonstrated efficacy in degrading neutrophil extracellular traps (85). Intestinal oxidative damage is prominent in both forms of IBD (86–88), and we have previously shown that deletion of *Socs3* in myeloid cells leads to elevated neutrophil activation and increased production of reactive oxygen species (44). Therefore, further investigation will be conducted to elucidate the mechanisms behind neutrophil-specific inflammatory responses in this model.

In summary, our study using a clinically relevant experimental colitis model provides further insights into the role of neutrophils in IBDs. Not only do these results unveil the potential for exploitation of neutrophil function, but they also demonstrate how targeting neutrophil activity holds potential clinical significance. As such, this may pave the way for therapies aimed at mitigating the effects of pro-inflammatory neutrophils.

## Data availability statement

The original contributions presented in the study are included in the article/**Supplementary Materials**, further inquiries can be directed to the corresponding author/s.

## Ethics statement

The animal study was reviewed and approved by University of Alabama at Birmingham.

## Author contributions

HQ contributes to experimental designs, results and data analysis and manuscript writing. ZY contributes to experimental designs, experiment performance, results and data analysis and manuscript writing. LZ contributes to experimental designs, experiment performance, results and data analysis and manuscript writing. WY contributes to experimental designs and experiment performance. JB contributes to experiment performance. SD contributes to Histology data analysis. EB contributes to experimental designs and manuscript writing.
