# Supplementary material for: Socs3 expression in myeloid cells modulates the pathogenesis of dextran sulfate sodium (DSS)-induced colitis
Source: Front Immunol. 2023 May 22;14:1163987. doi: 10.3389/fimmu.2023.1163987 (PMC10239850; doi:10.3389/fimmu.2023.1163987)
Supplement: Supplementary file 1 [file DataSheet_1.docx]

Zhou, L. et al., Supplementary Figure 1


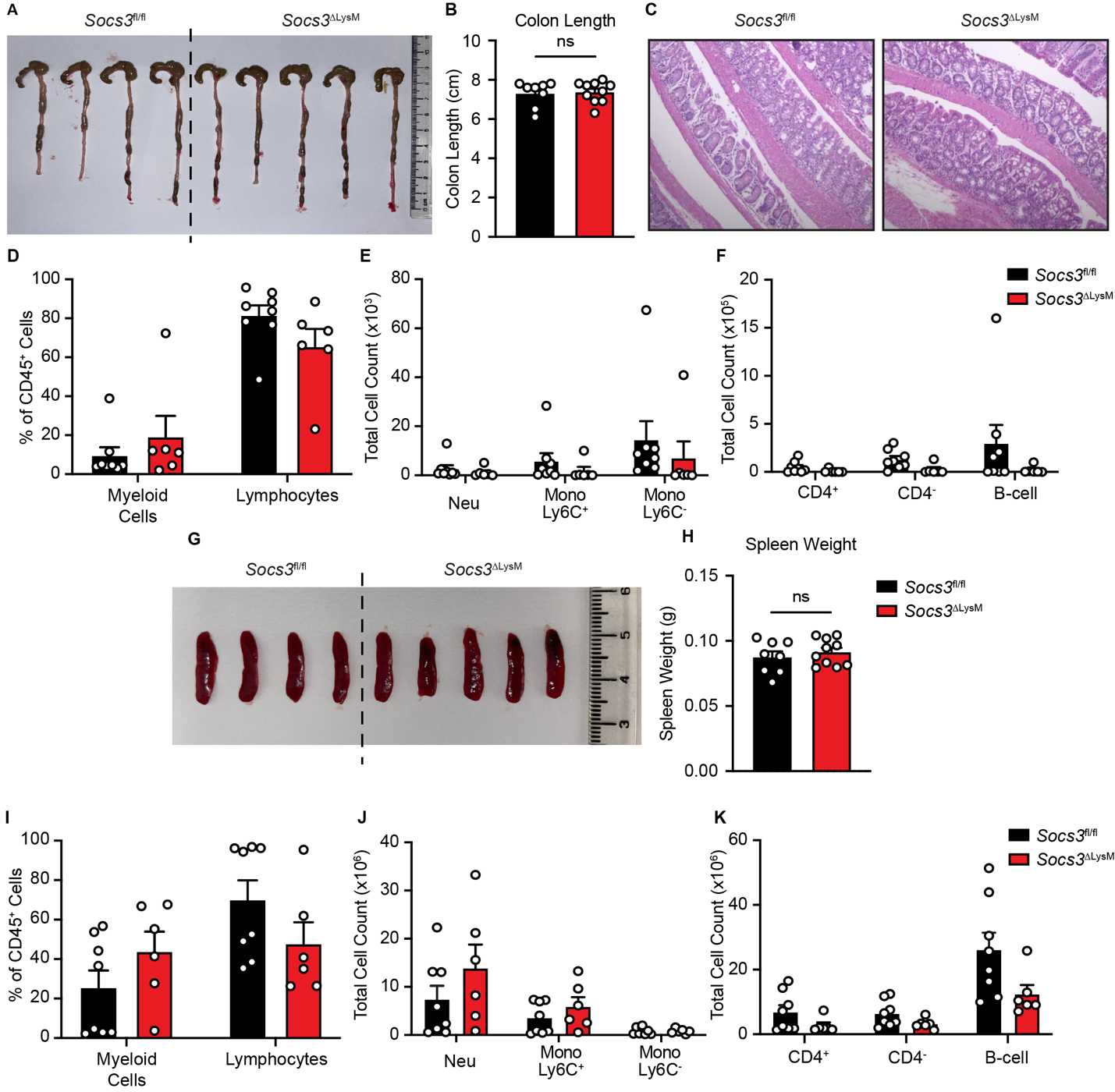


**Supplementary Figure 1. Comparable histology and baseline immune composition in the spleen and colon between *Socs3*^fl/fl^ and *Socs3*^ΔLysM^ mice.** Naïve *Socs3*^fl/fl^ (n = 8) and *Socs3*^ΔLysM^ (n = 10) mice were euthanized at baseline. **(A)** Representative pictures of the colon. **(B)** Statistics of colon length. **(C)** H&E staining of the distal colon. Relative frequency of immune cells (**D**) and total number of myeloid cells (neutrophils and monocyte subsets) **(E)** and lymphocytes (CD4^+^ T-cells, CD4^-^ T-cells, and CD19^+^ B-cells) **(F)** in the colon from *Socs3*^fl/fl^ (n = 8) or *Socs3*^ΔLysM^ mice (n = 6). **(G)** Representative pictures of the spleen. **(H)** Statistics of spleen weight. Relative frequency of immune cells (**I**) and total number of myeloid cells (neutrophils and monocyte subsets **(J)** and lymphocytes (CD4^+^ T-cells, CD4^-^ T-cells, and CD19^+^ B-cells) **(K)** in the spleen from *Socs3*^fl/fl^ (n = 8) or *Socs3*^ΔLysM^ mice (n = 6).

Zhou, L. et al., Supplementary Figure 2


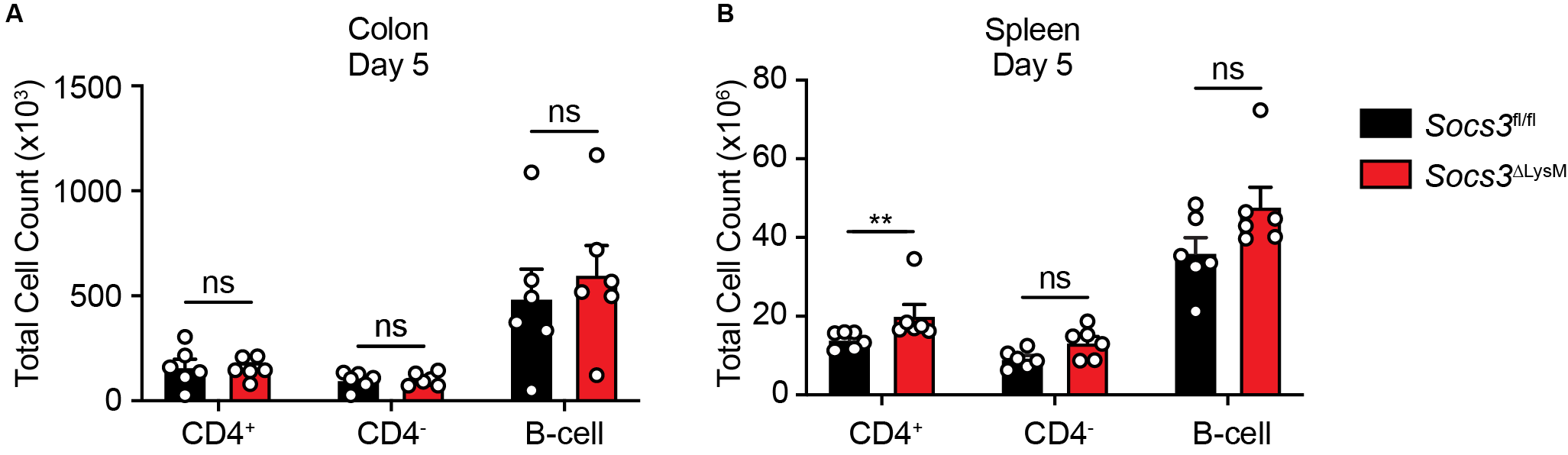


**Supplementary Figure 2. Lymphocyte counts in the spleen and colon in DSS-induced colitis.** *Socs3*^fl/fl^ (n = 6) or *Socs3* ^ΔLysM^ (n = 6) mice were fed with 3% DSS and euthanized on day 5. Cell numbers of CD4^+^ T-cells, CD4^-^ T-cells, and CD19^+^ B-cells in the colon **(A)** and spleen **(B)**. **p < 0.01.

Zhou, L. et al., Supplementary Figure 3


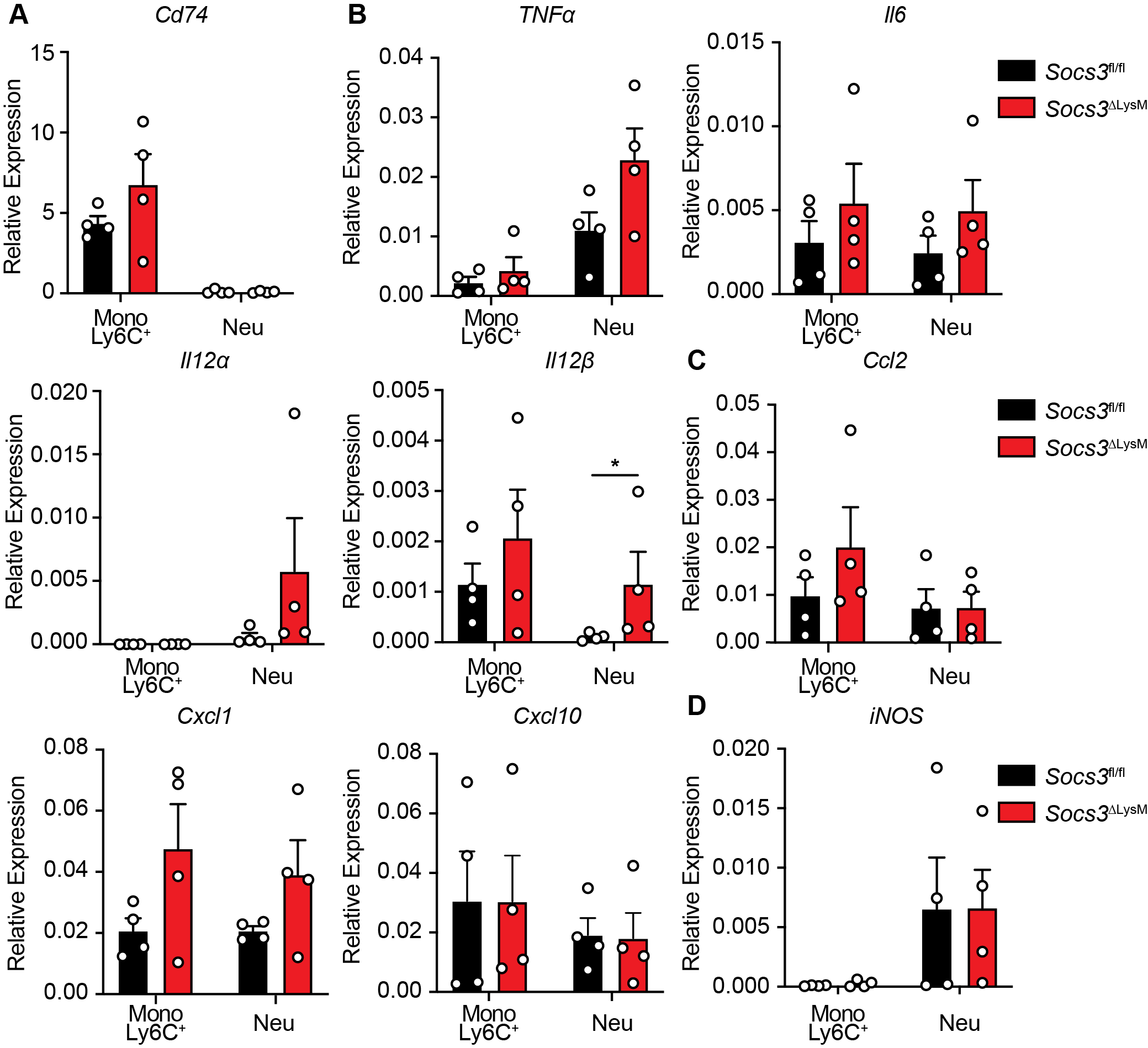


**Supplementary Figure 3. Expression of monocyte-related inflammatory genes in colonic myeloid cells in colitis.** *Socs3*^fl/fl^ (n = 8) or *Socs3* ^ΔLysM^ (n = 8) mice were fed with 3% DSS. Mice were euthanized on day 5, and immune cells were sorted from colon tissue by flow cytometry. Gene expression in Ly6C^+^ monocytes and neutrophils from *Socs3*^fl/fl^ or *Socs3* ^ΔLysM^ mice was examined. Gene expression of *Cd74* **(A)**, cytokines *TNF*$\alpha$*, Il6, Il12*$\alpha$*, Il12*$\beta$ **(B)**, chemokines *Ccl2, Cxcl1, Cxcl10* **(C)**, and *iNOS* **(D)** were determined by qRT-PCR, using 18s rRNA as an internal control. Two mice were combined for each experiment with 4 experiments in each group. *p < 0.05.

Zhou, L. et al., Supplementary Figure 4


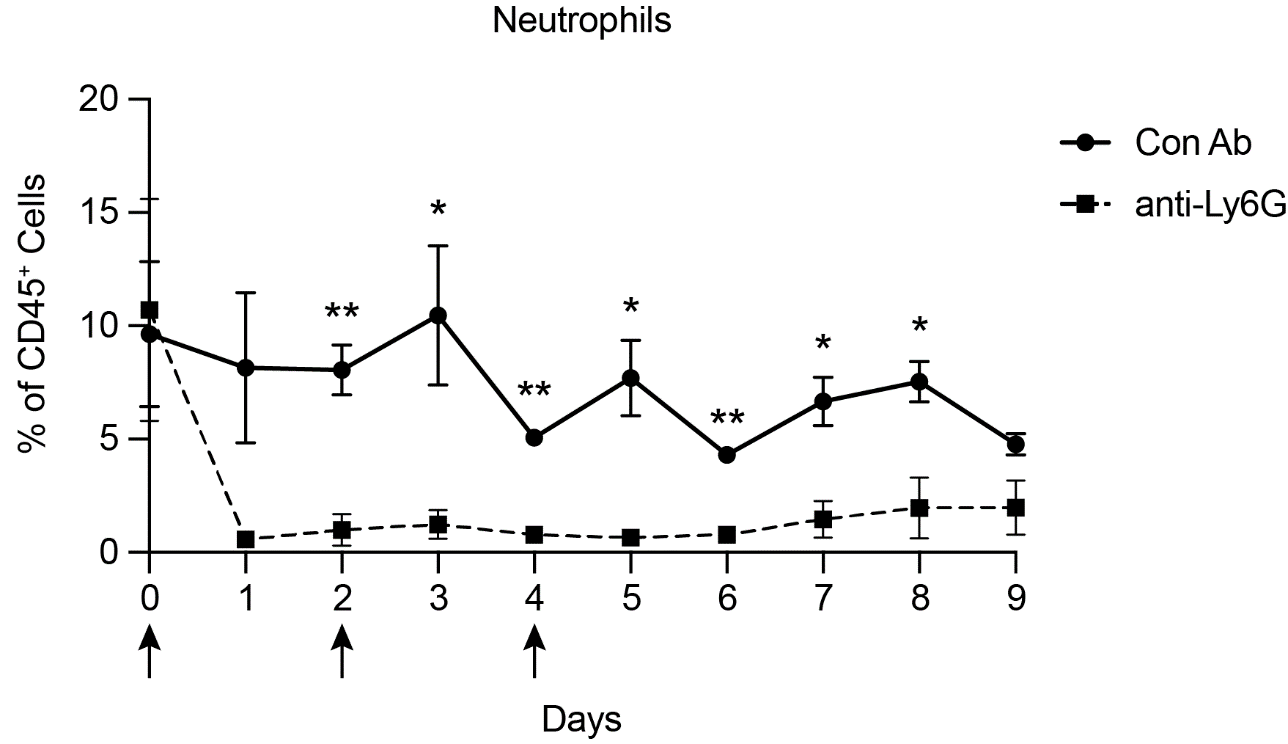


**Supplementary Figure 4. Significant reduction of circulating neutrophils after neutrophil-depletion regimen.** *Socs3*^fl/fl^ mice were injected with either anti-Ly6G or IgG2a (Con Ab) (n = 3) Abs (100 μg/dose) intraperitoneally at days 0, 2, and 4. Mice were bled each day. Cells from the peripheral blood were stained for multicolor flow cytometry analysis. Percentages of neutrophils were compared between the anti-Ly6G group and Con Ab group. *p < 0.05, **p < 0.01.

Zhou, L. et al., Supplementary Figure 5


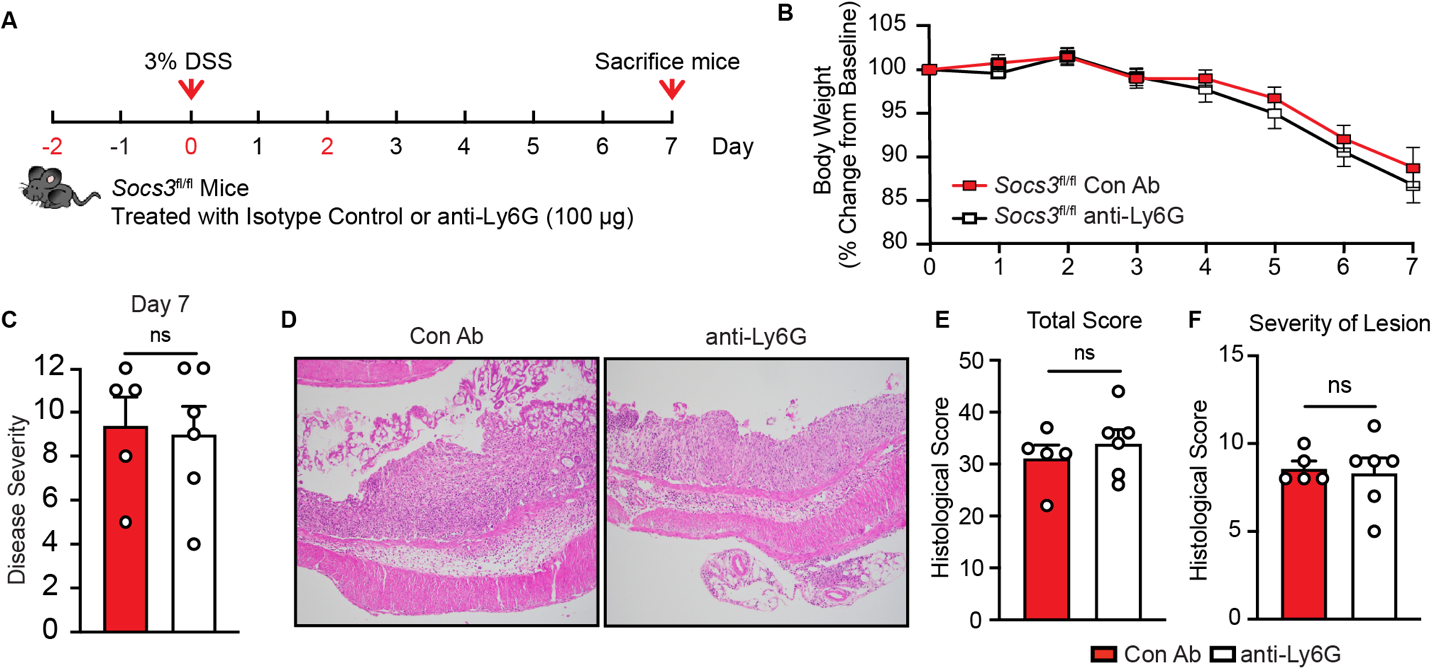


**Supplementary Figure 5. Neutrophil depletion does not ameliorate DSS-induced colitis in *Soc3*^fl/fl^ mice**. **(A)** Female *Soc3*^fl/fl^ mice were injected with either anti-Ly6G (anti-Ly6G; n = 6) or Isotype Control (Con Ab; n = 5) Abs (100 μg/dose) intraperitoneally at days -2, 0 and 2 (shown in red)*.* Mice were fed with 3% DSS for 7 days. **(B)** Mice were weighed daily, and weight loss was determined by comparing to day 0 for 7 days. **(C)** Disease severity on day 7. **(D)** H&E staining of the distal colon. **(E)** Total pathological scores of colons are shown using severity of lesion, degree of hyperplasia, degree of ulceration and percent of area involved. **(F)** Severity of lesion was calculated in *Soc3*^fl/fl^ mice administered anti-Ly6G or isotype control abs.
